# Human forced expiratory noise. Origin, apparatus and possible diagnostic applicationsa)

**DOI:** 10.1121/10.0002705

**Published:** 2020-12-01

**Authors:** Vladimir I. Korenbaum, Irina A. Pochekutova, Anatoly E. Kostiv, Veronika V. Malaeva, Maria A. Safronova, Oksana I. Kabantsova, Svetlana N. Shin

**Affiliations:** Pacific Oceanological Institute, Russian Academy of Sciences, 43 Baltiiskaya str., Vladivostok 690041, Russia

## Abstract

Forced expiratory (FE) noise is a powerful bioacoustic signal containing information on human lung biomechanics. FE noise is attributed to a broadband part and narrowband components—forced expiratory wheezes (FEWs). FE respiratory noise is composed by acoustic and hydrodynamic mechanisms. An origin of the most powerful mid-frequency FEWs (400–600 Hz) is associated with the 0th–3rd levels of bronchial tree in terms of Weibel [(2009). Swiss Med. Wkly. **139**(27–28), 375–386], whereas high-frequency FEWs (above 600 Hz) are attributed to the 2nd–6th levels of bronchial tree. The laboratory prototype of the apparatus is developed, which includes the electret microphone sensor with stethoscope head, a laptop with external sound card, and specially developed software. An analysis of signals by the new method, including FE time in the range from 200 to 2000 Hz and band-pass durations and energies in the 200-Hz bands evaluation, is applied instead of FEWs direct measures. It is demonstrated experimentally that developed FE acoustic parameters correspond to basic indices of lung function evaluated by spirometry and body plethysmography and may be even more sensitive to some respiratory deviations. According to preliminary experimental results, the developed technique may be considered as a promising instrument for acoustic monitoring human lung function in extreme conditions, including diving and space flights. The developed technique eliminates the contact of the sensor with the human oral cavity, which is characteristic for spirometry and body plethysmography. It reduces the risk of respiratory cross-contamination, especially during outpatient and field examinations, and may be especially relevant in the context of the COVID-19 pandemic.

## I. INTRODUCTION

Assessing human lung ventilation function is a standard approach for screening and diagnosing respiratory diseases, as well as monitoring effects of various extreme factors on the respiratory system, including diving, space flights, etc.

The common method of assessing human lung ventilation function is the flow-volume technique (spirometry and body plethysmography). Although filters and disposable or sterilized replaceable mouthpieces are used in the measuring equipment for testing pulmonary function, the risk of microbial contamination and cross-infection remains in this technique. It causes special restrictions on usage in current high-risk airborne respiratory infection during the COVID-19 pandemic (ERS, 2020). Moreover, the sensitivity of the traditional flow-volume technique of lung function diagnostics does not satisfy medical practice anymore. That is why attempts to develop alternative techniques for assessing lung ventilation function do not stop.

One of these attempts is connected to acoustic methods. Despite most of the studies in the field of respiratory acoustics being focused on the objectification of lung auscultation (see Rao *et al.*, 2018; D'yachenko and Mikhaylovskaya, 2012), there are very few works aimed at developing acoustic methods for assessing lung ventilation function. A special breathing maneuver of forced exhalation, i.e., maximal sharp and complete exhalation after a full inspiration, similar to that performed during spirometry, is used frequently in these works.

Forced expiratory (FE) noise is a bioacoustic signal containing information on biomechanics of lungs (see, for example, Lyubimov *et al.*, 2013). Furthermore, FE noise is characterized by high intensity and, consequently, a significantly higher signal-to-noise ratio than quiet breathing noise commonly used in medical auscultation of lungs. FE noise manifests via a powerful broadband part as well as by intensive narrowband components (see Korenbaum *et al.*, 1998). The narrowband components were previously named as forced expiratory wheezes (FEWs) by Forgacs (1978).

Due to the mentioned reasons, FE noise is actively investigated for diagnostic purposes. An assumption about the possibilities of objectively analysing FEWs for diagnostics of bronchial obstruction was originally hypothesized by Forgacs (1978). Ishikawa *et al.* (1992) subsequently compared tracheal FEWs of 15 healthy individuals and 15 patients with asthma and found that sounds of healthy persons had an energy maximum in the frequency range from 300 to 500 Hz. However, in asthma patients, a second spectral peak occurred in the frequency range between 1.3 and 1.5 kHz. Fiz *et al.* (2002) proposed the algorithm for automatic detection of FEWs in the three-dimensional spectrogram based on the results of the study by Shabtai-Musih *et al.* (1992). Fiz *et al.* (2006) demonstrated significant differences in the number of FEWs between asthma patients and healthy persons. However, diagnostic value of this parameter was insufficient. Korenbaum *et al.* (1998) also tried to use mid-frequency and high-frequency FEWs for diagnostics of asthma and chronic obstructive pulmonary disease (COPD); however, isolated evaluation of FEWs was found to be diagnostically ineffective.

Several authors have analysed FEWs in healthy individuals. Charbonneau *et al.* (1987) found that FEWs in healthy persons appeared after achievement of maximum flow velocity, but sometimes with essential delay. FEWs were also associated with certain forms of the flow-volume curve. Beck and Gavriely (1990) showed that FEWs registered above the trachea in healthy individuals were produced in 95% of all maneuvers. Moreover, in various maneuvers of each person through a tube of the same diameter (i.e., in identical flow conditions), the main component of the FEWs was characterized by a similar peak frequency in all attempts.

Nevertheless, the mechanisms of FE noise production and the levels of bronchial tree responsible for these noise origins, as well as possibilities to use parameters of FE noise for diagnosis and monitoring of human lung function, are still being discussed.

The objective of this work is to clarify acoustic considerations about the origin and characteristics of FE noises registered over trachea, to develop an acoustic technique for registering and processing FE noises, and to draft possible applications of this technique for diagnostics and monitoring of human lung function.

## II. ACOUSTIC CONSIDERATIONS ON ORIGIN OF FE NOISES

**FIG. 1.**
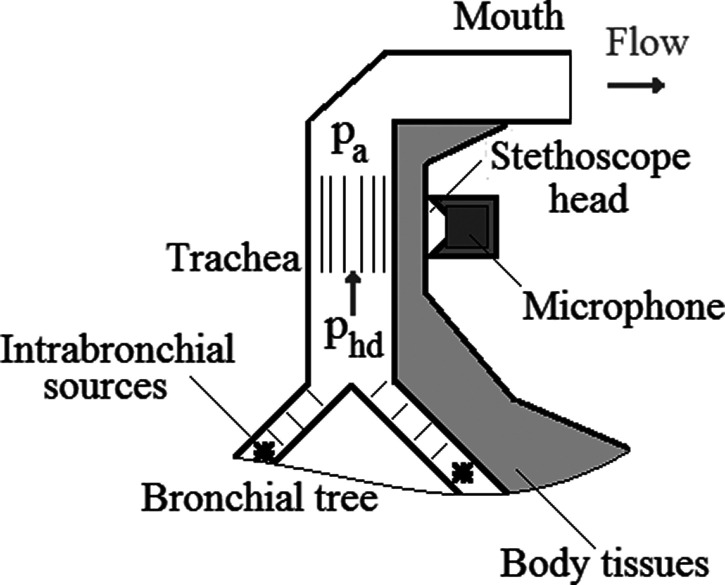
A scheme of FE noise emission.

A simplified scheme demonstrating FE signal origin and its recording above human trachea is shown in Fig. 1. During sound signal registration by a microphone, equipped with a stethoscope head, FE tracheal noises may appear in two ways (see D'yachenko *et al.*, 2012; Glazova *et al.*, 2018). The first one is a superposition of acoustic noises emitted into a lumen of airways. This mechanism involves a propagation of acoustic signals from distant intrabronchial sources (Fig. 1) through airways of the bronchial tree, resulting in formation of an acoustic pressure (*p_a_*) inside the trachea lumen. The second mechanism is hydrodynamic or so-called pseudo-sound effect of turbulent pressure pulsations of airflow vortices to the tracheal inner wall. This effect results in an averaged hydrodynamic pressure (*p_hd_*). Unlike the first (acoustic) mechanism, there is no need for air medium compressibility here, and the ecorded signal is proportional to the hydrodynamic pressure in the turbulent flow averaged through an area of perception of the acoustic sensor. Due to the physics of its operation, the stethoscope sensor on the neck does not distinguish these mechanisms of pressure changes on the tracheal inner wall and registers equally both signal components outside trachea (see Korenbaum *et al.*, 2016b).

**FIG. 2.**
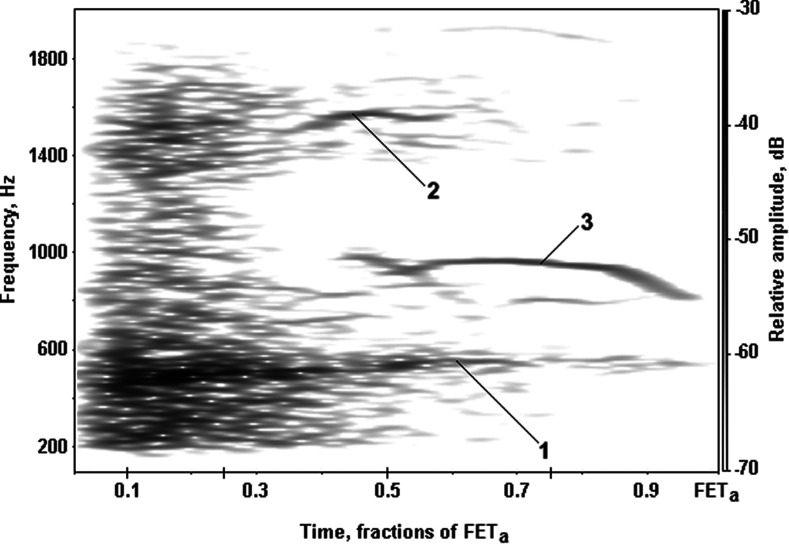
The spectrogram of FE tracheal noises of the healthy subject, recorded on trachea by the electret microphone with stethoscope head described later: 1, mid-frequency FEW; 2, early high-frequency FEW; 3, late high-frequency FEW.

As one can see in the spectrogram (Fig. 2), FE noises are represented by the powerful broadband part and intensive narrowband FEWs. According to the spectrogram (Fig. 2), off-prints of some types of FEWs may be identified. The first one—a path of the most prominent mid-frequency FEW seen usually during almost all time of FE maneuver in the frequency range from 400 to 600 Hz (1), the second one—a path of the early high-frequency FEW seen in the first half of FE maneuver in the frequency range over 600 Hz (2), the third one—a path of the late high-frequency FEW seen in the second half of FE maneuver in the frequency range over 600 Hz. Although peak frequencies of FEWs vary essentially between subjects, the scheme of FEWs appearance, described in Fig. 2 may be found above trachea in the majority of volunteers and patients (Korenbaum *et al.*, 2013b).

Korenbaum *et al.* (1997) and Korenbaum *et al.* (2009) supposed that FEWs may be divided into sounds generated by flow-dependent mechanisms (vortices shedding, forced dynamic flutter) and flow-independent self-oscillatory mechanisms (self-oscillatory flutter or oscillations in the closing of mucous tissue). An origin of the most powerful mid-frequency FEWs (400–600 Hz) was associated with the 0th–3rd levels of branching of the bronchial tree according to the Weibel (2009) scheme, whereas high-frequency FEWs (above 600 Hz) were attributed to the 2nd–6th levels of bronchial tree branching (see Korenbaum *et al.*, 2013b).

Thus a variety of identified acoustic FE effects opens new opportunities for diagnostic applications.

## III. METHOD AND APPARATUS

The acoustic method of diagnostics of lung function is developed. It is based on the estimation of noise parameters of human forced exhalation, recorded above trachea (Fig. 1). As assessing FEWs parameters turned out to be technically difficult, alternative band-pass acoustic parameters of the FE noise were developed. Taking into account a dependence of FE noise amplitude characteristics on properties of acoustic tracts, special attention was paid to develop parameters independent of the sensitivity of sensors and amplification of electronics.

The first parameter suggested was FE noise time in the frequency range of 200–2000 Hz (FET_a_), which characterized the noise process in common. A frequency band below 200 Hz was excluded due to high level of interference associated with the vibration of an acoustic sensor when touching soft tissues. A frequency band above 2000 Hz was excluded due to the low level of the signal. The FET_a_ is less dependent on properties of acoustic tracts than any amplitude characteristics.

Measurement of FET_a_ for each recorded file was taken by using a specially developed algorithm. Filtration was carried out in the frequency band of 200–2000 Hz (Kaiser windowed direct-form finite impulse response, FIR, filter). The FE waveform envelope was constructed doubly in the forward and opposite directions by moving the average method with an accumulation period of 0.01 s. Then the peak amplitude (*U*) of the envelope was calculated. The threshold level *S* = 0.005*U* was defined. The times of beginning *T*_1_ and ending *T*_2_ of the FE noise process were measured by the threshold level *S* of the envelope when moving from the peak to the left and to the right. Since *T*_1_, *T*_2_ were measured automatically or semiautomatically (see Korenbaum *et al.*, 2013a), the program automatically calculated the FE noise time as the difference FET_a_ = *T*_2_ – *T*_1_. The maximal individual FET_a_ from at least three well-done attempts of forced exhalations was used for further analysis.

Additionally, the band-pass durations and energies in the bands of 200 Hz lying inside the total frequency range 200–2000 Hz are developed supposedly more suitable for estimating narrow-band FE noise parameters than FEWs. They are specified in Table I.

**TABLE I.** The list of developed band-pass energies and durations in the 200 Hz bands, where A = ΣA_i_.

*i*	Frequency band, Hz	*A_i_*, conventional unit	*t_i_*, second	*Ar_i_*, fraction of 1	*tr_i_*, fraction of 1
1	200–400	*A*_200–400_	*t*_200–400_	*A*_200–400_/*A*	*t*_200–400_/FET_a_
2	400–600	*A*_400–600_	*t*_400–600_	*A*_400–600_/*A*	*t*_400–600_/FET_a_
3	600–800	*A*_600–800_	*t*_600–800_	*A*_600–800_/*A*	*t*_600–800_/FET_a_
4	800–1000	*A*_800–1000_	*t*_800–1000_	*A*_800–1000_/*A*	*t*_800–1000_/FET_a_
5	1000–1200	*A*_1000–1200_	*t*_1000–1200_	*A*_1000–1200_/*A*	*t*_1000–1200_/FET_a_
6	1200–1400	*A*_1200–1400_	*t*_1200–1400_	*A*_1200–1400_/*A*	*t*_1200–1400_/FET_a_
7	1400–1600	*A*_1400–1600_	*t*_1400–1600_	*A*_1400–1600_/*A*	*t*_1400–1600_/FET_a_
8	1600–1800	*A*_1600–1800_	*t*_1600–1800_	*A*_1600–1800_/*A*	*t*_1600–1800_/FET_a_
9	1800–2000	*A*_1800–2000_	*t*_1800–2000_	*A*_1800–2000_/*A*	*t*_1800–2000_/FET_a_

**FIG. 3.**
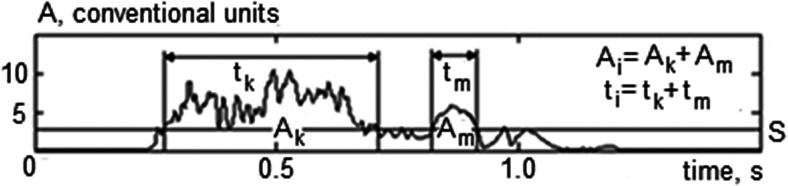
Example of evaluating the band-pass duration *t_i_* and the band-pass energy *A_i_* by the envelope of FE noise signal filtered in the *i*th 200-Hz band.

The special algorithm is designed to calculate *A_i_* and *t_i_* shown as an example in Fig. 3.

The band-pass durations are measured using the envelope of the signal filtered in each 200-Hz frequency band, constructed by moving average method, by means of summing the number of all time samples of the signal in which amplitudes exceeded the threshold *S*. To evaluate the band-pass durations, the number of fixed time samples is multiplied by the period between two samples of the envelope (0.01 s). The band-pass energies are calculated using the same envelope. Here, the amplitudes of fixed time samples of the signal are summed if their magnitudes exceed the threshold *S*. The sections of the envelope of the signal lying below the threshold *S* are excluded in both cases. Vice versa, all fragments of the envelope of the signal lying above the threshold *S* are summed as *A_i_* = *A_k_* + *A_m_*, and *t_i_* = *t_k_* + *t_m_* (Fig. 3). The threshold *S* = 0.005*U* is the same as used in FET_a_ measuring procedure.

Finally, normalized band-pass durations *tr_i_* = *t_i_*_/_FET_a_ and normalized band-pass energies *Ar_i_* = *A_i_*/*A* (*i* = 1,…,9) are calculated, where *A* = Σ*A_i_* is total energy it the frequency range of 200–2000 Hz. Note that normalized band-pass parameters, in contrast to absolute ones, are relative values, thus insensitive to variations in the transfer coefficient of sensors and amplifiers. It makes these acoustic parameters more convenient for diagnostic usage.

To illustrate typical values for the developed parameters in all 200-Hz bands as well as their repeatability in the form of the coefficient of variation, CV = SD/M, Table II is included, summarizing data for one healthy male subject collected in his four attempts of FE maneuver.

**TABLE II.** An example of a data set of the healthy male subject averaged in four attempts of FE maneuver.

Parameter	Units	*M*	CV,%
FET_a_	s	1.441	1.7
*t*_200–400_	s	1.121	4.5
*t*_400–600_	s	1.262	2.5
*t*_600–800_	s	1.180	9.1
t_800–1000_	s	1.246	6.7
*t*_1000–1200_	s	1.325	5.0
*t*_1200–1400_	s	1.185	1.6
*t*_1400–1600_	s	0.807	17.2
*t*_1600–1800_	s	0.691	31.0
*t*_1800–2000_	s	0.575	11.0
*A*_200–400_	Conventional	19.082	43.6
*A*_400–600_	Conventional	19.711	32.7
*A*_600–800_	Conventional	14.092	40.9
*A*_800–1000_	Conventional	25.198	39.5
*A*_1000–1200_	Conventional	31.651	30.3
*A*_1200–1400_	Conventional	17.786	24.4
*A*_1400–1600_	Conventional	5.362	38.6
*A*_1600–1800_	Conventional	3.196	29.0
*A*_1800–2000_	Conventional	1.714	25.6
*tr*_200–400_	Fractions of 1	0.778	4.5
*tr*_400–600_	Fractions of 1	0.876	2.5
*tr*_600–800_	Fractions of 1	0.819	8.6
*tr*_800–1000_	Fractions of 1	0.865	7.4
*t*_1000–1200_	Fractions of 1	0.919	5.0
*tr*_1200–1400_	Fractions of 1	0.823	2.6
*tr*_1400–1600_	Fractions of 1	0.559	16.0
*tr*_1600–1800_	Fractions of 1	0.478	30.3
*tr*_1800–2000_	Fractions of 1	0.399	10.2
*Ar*_200–400_	Fractions of 1	0.137	15.6
*Ar*_400–600_	Fractions of 1	0.148	11.0
*Ar*_600–800_	Fractions of 1	0.103	13.3
*Ar*_800–1000_	Fractions of 1	0.183	15.3
*Ar*_1000–1200_	Fractions of 1	0.241	17.9
*Ar*_1200–1400_	Fractions of 1	0.136	12.2
*Ar*_1400–1600_	Fractions of 1	0.040	13.7
*Ar*_1600–1800_	Fractions of 1	0.024	16.5
*Ar*_1800–2000_	Fractions of 1	0.013	20.1

The laboratory prototype of apparatus is developed, which includes an acoustic sensor–electret microphone (W62A) with an ebonite stethoscope head. The head has a conical chamber of 20 mm diameter at its base and 5 mm in depth (opening angle of 120°). A laptop provided with an external sound card Transit (M-Audio) and specially developed software is used to introduce signal from the microphone and to evaluate FET_a_ (see Korenbaum *et al.*, 2013a) and the 200-Hz band-pass durations and energies in an automatic manner.

During measurements, the sensor is attached to the lateral neck surface and the subject or physician (operator) holds the box with his hand pressing a stethoscope from head to the body. Nose-clamp is used. The subject performs a forced exhalation maneuver from maximal inspiration. A delay of about 0.5 s is made between inspiration and exhalation. In order to carry out the maneuver properly, a maximum sharp and complete exhalation is required (see Pochekutova and Korenbaum, 2013).

To assess possible diagnostic applications of the developed acoustic method, let us analyse now the results of processing FE noise signals previously collected by our team in volunteers and patients.

## IV. RESULTS AND DISCUSSION OF SOME DIAGNOSTIC APPLICATIONS

The first question is whether the developed acoustic parameters reflect traditional biomechanical flow-volume indices of human pulmonary function.

The main method of study of acoustical-biomechanical relations was a comparison of the FE tracheal noise acoustic parameters with the results of the evaluation of biomechanical indicators of human lung function, which were obtained not only by means of spirometry but also with body plethysmography (MasterScreen Body, Jager).

**FIG. 4.**
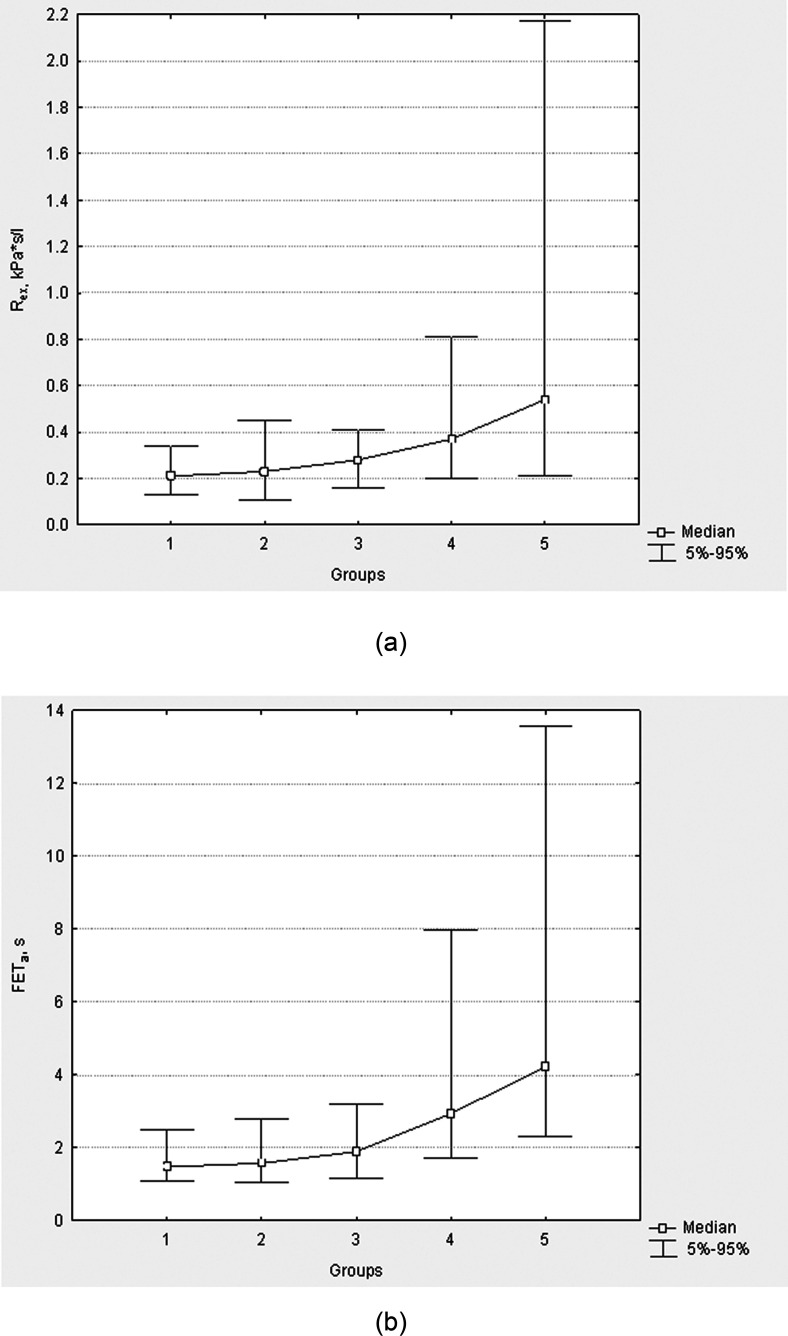
Diagrams of the dependence of body plethysmographic airway resistance *R_ex_* (a), and FET_a_ (b) on the “incidence and severity” of bronchial obstruction in specific groups of the sample of 218 persons (Jonckheere-Terpstra test, *p* < 0.001 both for *R_ex_* and FET_a_); 1, healthy volunteers, 2, subjects with risk factors of bronchial obstruction; 3, spirometry negative asthma patients; 4, spirometry positive asthma patients; 5, COPD patients.

Korenbaum *et al.* (2016a) studied the sample consisting of 218 volunteers, which included healthy subjects (*n* = 50), persons with risk factors of chronic respiratory diseases (*n* = 60), patients with spirometry negative (*n* = 32), and spirometry positive (*n* = 41) bronchial asthma and COPD (*n* = 35). It is evident from clinical considerations that the factor of “incidence and severity” of bronchial obstruction increases gradually in the sample from the 1st group to the 5th one. By means of nonparametric Jonckheere-Terpstra analysis of variance (ANOVA) analysis, a statistically significant (*p* < 0.001) effect of the factor of “incidence and severity” of bronchial obstruction on lung function biomechanical indices is revealed, for example, on the body plethysmographic airway resistance [Fig. 4(a)]. Thus, now using the sample characterized by the significant gradual increase in the body plethysmographic airway resistance [Fig. 4(a)] as the model, one could see that FET_a_ [Fig. 4(b)] is well coordinated with the airway resistance (Jonckheere-Terpstra test, *p* < 0.001). This observation confirms previously developed model considerations of FE noise production in healthy individuals, in individuals with bronchial obstruction (see Korenbaum and Pochekutova, 2008), and in volunteers breathing gases of various density (see D'yachenko *et al.*, 2012), connecting FET_a_ with indirect measures of FE airway resistance.

Furthermore, Pochekutova and Korenbaum (2013) demonstrated for the FET_a_ acoustic parameter a sufficiently effective acoustic diagnosis of bronchial obstruction in patients with spirometry confirmed asthma (group 4 in Fig. 4) with sensitivity and specificity of about 90%, as well as a possibility of detection of hidden bronchial obstruction not revealed by traditional spirometry in near 50% of the group 3 (Fig. 4) with spirometry negative asthma.

Malaeva *et al.* (2017) revealed significant interrelations between band-pass acoustic durations and energies of the FE tracheal noises and biomechanical indices of lung function by means of nonparametric Spearman correlation analysis in the sample. The strongest correlation coefficients are noted between band-pass durations and measures of airway resistances (up to 0.59), reflecting primarily the function of large airways, as well as the residual volume of the lungs (up to 0.47) and its ratio to the total lung capacity (up to 0.43) that characterize the state of small airways. The significant bidirectional correlation between acoustic FE tracheal parameters and biomechanical indices were additionally revealed in each specific group of the sample.

The analyzed findings mean that developed FE acoustic parameters correspond to basic indices of human lung function measured by spirometry and body plethysmography, and in some cases (for example, spirometry negative asthma), may be even more sensitive to respiratory deviations than mentioned indices.

To assess some possibilities of the acoustic estimation of the impact of various extreme factors to the human respiratory system, we studied diving submersions and postural modeling of microgravity.

Pochekutova and Korenbaum (2011) applied the developed acoustic technique to divers (48 subjects) and revealed an increase of FET_a_ interpreted as the acoustic sign of transient bronchial obstruction features in 13 subjects (27%) after single shallow-water sea submersion with the old fashioned closed-circuit breathing apparatus IDA-71 (SU). The effect was probably caused by the development of inflammation of bronchial mucosa and accompanying edema due to the toxic effect of hyperbaric hyperoxia in combination with small doses of the regenerative substance (including caustic potassium alkali) vapor. These signs of toxic damage to the pulmonary system appeared in time intervals not exceeding the permissible period of the diving operation with oxygen. The observation dictated a necessity to provide individual control of divers' lung function during the training process in a closed-circuit breathing apparatus in order to prevent accidents and to achieve professional longevity.

It is supposed that a modern closed-circuit breathing apparatus would not have such influence on human lung function due to an absence of the regenerative substance, including caustic potassium alkali. To test this supposition, a group of 25 male divers performed single shallow-water sea submersion (less than 1 h) in a modern closed-circuit breathing apparatus FROGS (AquaLung, France) (see Table III).

**TABLE III.** The significance (*p*) of differences of the FE acoustic parameters in divers before and after underwater submersion and in healthy volunteers before and after medical bronchodilator test according to Wilcoxon *t*-test. ns, *p* > 0.05; (−), significant (*p* < 0.05) decrease in the parameter; (+), significant (*p* < 0.05) increase in the parameter.

Studied parameter	Underwater submersion (*n* = 25)	Medical bronchodilator test (*n* = 29)
FET_a_	ns	ns
*Ar*_1400–1600_	0.03 (−)	ns
*Ar*_1600–1800_	ns	0.017 (+)
*Ar*_1800–2000_	ns	ns

Although there is no significant rise of FET_a_ in relation to background status, a significant decrease of normalized band-pass high-frequency energy *Ar*_1400–1600_ is revealed (Wilcoxon *p* = 0.03). It is interesting that in the referent group of healthy volunteers (*n* = 29), consisting of 16 males and 13 females under a medical salbutamol bronchodilator test (Table III), the opposite significant response of the adjacent normalized band-pass high-frequency energy *Ar*_1600–1800_ is observed (Wilcoxon *p* = 0.017). Thus the response found in divers may be treated as an adverse influence of even a short oxygen submersion on increasing airway resistance. This effect is consistent with those obtained for the closed-circuit breathing apparatus of the previous generation (Pochekutova and Korenbaum, 2011). It looks like the phenomenon revealed does not depend on the configuration of the closed-circuit breathing apparatus, but is rather defined by hyperbaric hyperoxia itself. This conclusion seems to coordinate well with the results of a recent study by Wingelaar *et al.* (2019), which involved comparative analysis of volatile organic compounds in exhaled air after one-hour underwater dive using air and oxygen. In oxygen divers, a significant increase in the content of volatile compounds (methyl alkanes) is revealed, which is connected with damage of phosphatidylcholine membranes of pulmonary structures by hyperbaric hyperoxia. Nevertheless, flow-volume indices of spirometry have not changed yet. Therefore, our current study found that acoustic signs of an increase in airway resistance may be treated as a result of similar damage/inflammation reaction in the human respiratory tract.

As for microgravity simulation, Malaeva *et al.* (2018) previously studied postural simulation of microgravity by means of head-down −6° test (exp_1, five males) and lunar gravity by means of head-up +9.6° test (exp_2, six males) during a 20-day experiment. The reason for postural changes was a simulation of a lunar mission with part ofa crew in an orbital device (microgravity) and another part on the Moon's surface (lunar gravity). Both subgroups experienced five days in the head-down position (simulating space flight). On the fifth day, the second subgroup was transferred into the head-up position (simulating landing on the Moon). It was revealed that FET_a_ could distinguish subgroups subjected to these impacts from the 6th–20th days of the experiment (2-factor ANOVA), while there was no essential response of basic spirometry indices found. Furthermore, FET_a_ in prolonged simulation of microgravity was significantly higher than in prolonged simulation of lunar gravity. Using acoustical-biomechanical model considerations, an increase of FET_a_ in the microgravity model may be explained by an additional rise in airway resistance with respect to the lunar gravity model.

**FIG. 5.**
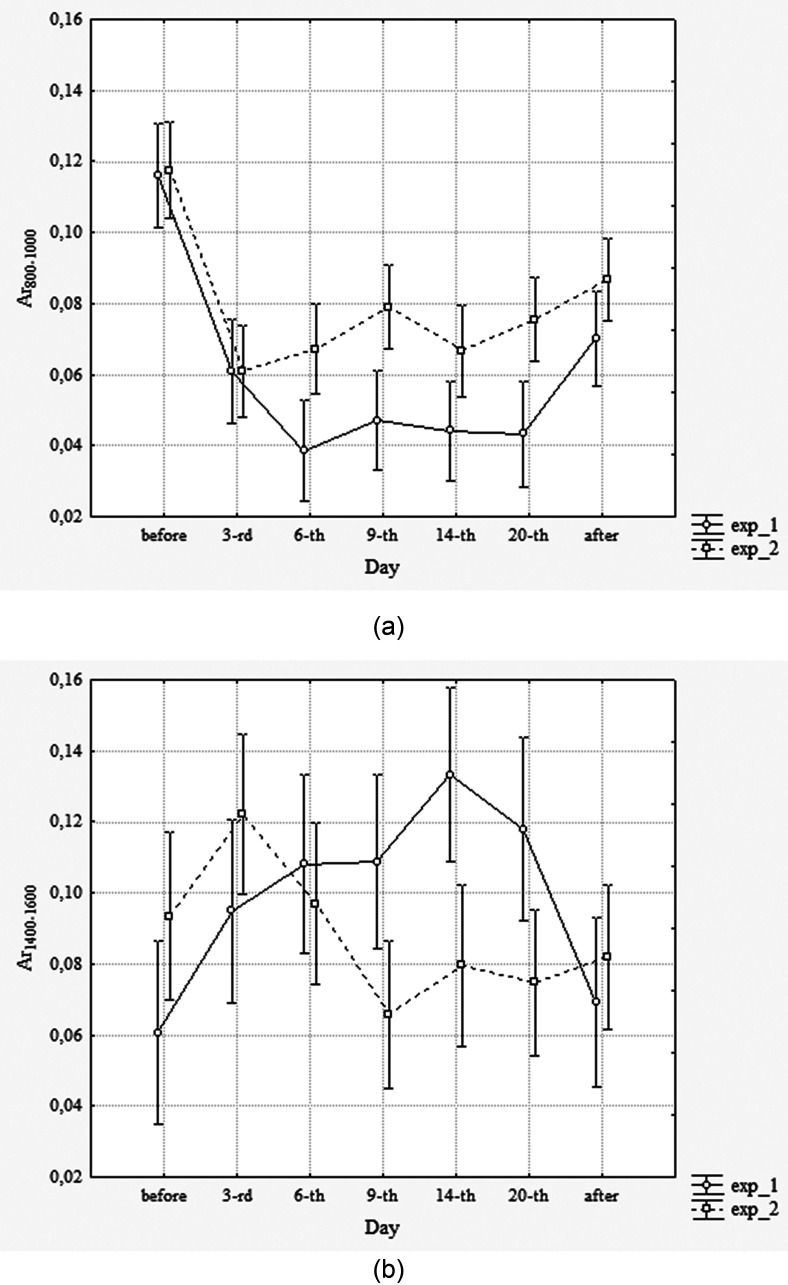
The diagrams of the normalized band-pass energies by days of the experiment of simulation microgravity (exp_1) and microgravity/lunar gravity (exp_2); (a) *Ar*_800–1000_, exp_type (*F*-test *p* < 0.001); (b) *Ar*_1400–1600_, exp_type*day (*F* test *p* < 0.001); means with 95% CI limits; circles, exp_1; squares, exp_2.

The developed normalized band-pass energies are applied additionally to analyze the results of that experiment. It is found that relative band-pass energies *Ar*_800–1000_ and *Ar*_1400–1600_ (Fig. 5) could distinguish studied subgroups (exp_1 vs exp_2) in the time interval the from 6th to 20th days of the experiment (2-factor ANOVA) no less successfully than FET_a_ in Malaeva *et al.* (2018).

It is interesting that directions of responses of these newly developed parameters to long-term postural effects are opposite (Fig. 5). Moreover, in accordance with the results of the Least Significant Difference (LSD)-test (Table IV), the first parameter significantly responds to postural change immediately on the sixth day of the experiment, while the second one reacts later—only by the ninth day of the experiment.

**TABLE IV.** Statistical significance (*p*) of distinctions of the normalized band-pass energies (Fig. 5) between the model of microgravity (exp_1) and model of microgravity/lunar gravity (exp_2) in the same days of the experiment according to LSD test. ns, *p* < 0.05.

	Before	3rd	6th	9th	14th	20th	After
*Ar*_800–1000_	ns	ns	0.004	0.0007	0.02	0.0009	ns
*Ar*_1400–1600_	ns	ns	ns	0.009	0.002	0.02	ns

These findings open the possibility to more detailed acoustic-biomechanical interpretation in studied postural models. On the other hand, the results seem promising for the implementation of subtle individual control of human pulmonary function in microgravity conditions.

Consequently, based on preliminary results of analyzed diagnostic applications, the developed technique may be considered a promising instrument for solving the problem of acoustic monitoring of human lung function status in various extreme conditions, including diving and space flights.

Additional studies connected to more extensive exploration of the developed set of FE acoustic parameters (Table I) in clinical testing of wider samples of volunteers and respiratory patients are welcome.

The developed technique has a sufficiently high sensitivity to detect deviations of human lung ventilation function. Its findings, as a rule, coordinate well with flow-volume pulmonary indices. However, the developed technique eliminates the contact of the sensor with the oral cavity, which is characteristic of spirometry and body plethysmography. It reduces the risk of respiratory cross-contamination, especially during outpatient and field examinations, and may be especially relevant in the context of the COVID-19 pandemic.

## V. CONCLUSIONS

An origin of FE noise is detailed in relation to mechanisms of the broadband part as well as narrowband FEWs. Informative acoustic parameters of FE noise, suitable for diagnostics of lung function, are found. The laboratory prototype of the apparatus is developed, which includes an electret microphone with stethoscope head, a laptop with an external sound card, and specially developed software. The FE time in the range of 200–2000 Hz and the band-pass durations and energies in 200 Hz bands are evaluated instead of FEW direct measures. It is demonstrated experimentally that developed FE acoustic parameters correspond to basic indices of lung function evaluated by spirometry and body plethysmography and may be even more sensitive to respiratory deviations. According to preliminary experimental results, the developed technique may be considered a promising instrument for acoustic monitoring of human lung function in extreme conditions, including diving and space flights. It is important that, in comparison with traditional flow-volume methods, the developed technique reduces the risk of respiratory cross-contamination.
